# Benefits of Binaural Integration in Cochlear Implant Patients with Single-Sided Deafness and Residual Hearing in the Implanted Ear

**DOI:** 10.3390/life11030265

**Published:** 2021-03-23

**Authors:** Artur Lorens, Anita Obrycka, Piotr Henryk Skarzynski, Henryk Skarzynski

**Affiliations:** 1 World Hearing Center, Implant and Auditory Perception Department, Institute of Physiology and Pathology of Hearing, 02-042 Warsaw, Poland; a.lorens@ifps.org.pl; 2 World Hearing Center, Teleaudiology and Screening Examination Department, Institute of Physiology and Pathology of Hearing, 02-042 Warsaw, Poland; p.skarzynski@ifps.org.pl; 3 World Hearing Center, Oto-Rhino-Laryngology Clinic, Institute of Physiology and Pathology of Hearing, 02-042 Warsaw, Poland; skarzynski.henryk@ifps.org.pl

**Keywords:** cochlear implant, single-sided deafness, hearing preservation, binaural effects, binaural integration

## Abstract

The purpose of the study is to gauge the benefits of binaural integration effects (redundancy and squelch) due to preserved low-frequency residual hearing in the implanted ear of cochlear implant users with single-sided deafness. There were 11 cochlear implant users (age 18–61 years old) who had preserved low-frequency hearing in the implanted ear; they had a normal hearing or mild hearing loss in the contralateral ear. Patients were tested with monosyllabic words, under different spatial locations of speech and noise and with the cochlear implant activated and deactivated, in two listening configurations—one in which low frequencies in the implanted ear were masked and another in which they were unmasked. We also investigated how cochlear implant benefit due to binaural integration depended on unaided sound localization ability. Patients benefited from the binaural integration effects of redundancy and squelch only in the unmasked condition. Pearson correlations between binaural integration effects and unaided sound localization error showed significance only for squelch (*r* = −0.67; *p* = 0.02). Hearing preservation after cochlear implantation has considerable benefits because the preserved low-frequency hearing in the implanted ear contributes to binaural integration, presumably through the preserved temporal fine structure.

## 1. Introduction

In cochlear implant (CI) users the advantage of binaural hearing over the monaural hearing has been extensively studied in terms of improvement in localization and speech discrimination over the monaural condition [1,2,3,4,5]. Until now, binaural benefits have usually been investigated under three binaural CI arrangements—bilateral, bimodal, and unilateral hearing loss. In the bilateral arrangement, two CIs are implanted, providing individuals who have severe-to-profound hearing loss with binaural auditory input. In the bimodal arrangement, and where residual hearing permits, a hearing aid is fitted to the non-implanted ear. In patients with unilateral hearing loss (UHL), the CI arrangement is different from the traditional bimodal one because patients with UHL have much more hearing in the non-implanted ear and this can have a significant impact on binaural benefits [1]. In the literature, two UHL populations have been defined—the first are patients with asymmetric hearing loss (AHL) who have profound hearing loss in one ear and mild-to-severe hearing loss in the other (according to the International Bureau for Audiophonology—BIAP classification); the second are patients with single-sided deafness (SSD) whose second ear has normal or close to normal hearing (NH) [6]. 

However, recent advances in the preservation of low-frequency (LF) hearing in the implanted ear give rise to another possible bilateral arrangement in patients with partial deafness [7,8]. Unilateral implant recipients who have preserved hearing in the implanted ear and are users of electric–acoustic stimulation (EAS) possess bilateral acoustic hearing at low frequencies, which can facilitate a binaural benefit [9]. In all these arrangements (except for bilateral implantation), there are different combinations of electric and acoustic hearing. These arrangements provide cues for binaural processing; therefore, in this way, they can provide possible binaural benefits. 

For these patients, the binaural benefit arises from listening to whichever ear has the better signal-to-noise ratio (the “better ear” effect) and from integrating information from both ears. The “better ear” effect depends on the head shadow effect, which provides different intensities to each ear. Listeners make use of the head shadow effect by listening with both ears and deciding which ear has the better SNR and ignoring the other. The “better ear” effect predominates at high frequencies [10,11]. 

Binaural redundancy and squelch rely on integrating information received from both ears. Binaural redundancy involves simultaneously processing two signals, one from each ear; since the same signal arrives at each ear with almost the same auditory characteristics, the brain can use overlapping information to better discriminate speech [12]. Redundancy can only be useful if sounds are audible in both ears. 

The squelch effect refers to the improvement in speech discrimination that occurs when a signal and the background noise are spatially separate; in this case, the added second input at the contralateral ear has a poorer signal-to-noise ratio than in the first ear. In order to take advantage of the squelch effect, it is essential for the central nervous system to have access to interaural differences between the received sounds, mainly the fine interaural time differences (ITD_fine_) conveyed through the temporal fine structure of the signal [10,11,13]. 

Binaural redundancy and binaural squelch predominate at low frequencies, and these two effects are often referred to as binaural integration effects. For the three binaural CI arrangements (bilateral, bimodal, and CI in unilateral hearing loss), one of the main limitations to binaural integration seems to arise from the limited temporal fine structure a cochlear implant can provide [14]. In the fourth arrangement (preserved LF hearing in the implanted ear), it can be assumed that implanted recipients would have significantly greater binaural integration compared to the other three arrangements because there is now access to fine time structure derived from bilateral acoustic low frequency (LF) hearing [15]. In this arrangement, access to fine structure in the implanted ear can improve speech performance in noise, facilitating better access to fundamental speech frequencies and providing ITD_fine_ when speech and signal are spatially separate [13,14,15,16]. 

The main goal of the present research is to evaluate the contribution of preserved LF hearing to binaural integration effects. Our hypothesis is that, due to hearing preservation in the implanted ear, bilateral acoustic LF hearing should provide extra hearing benefits via squelch and redundancy effects (over and above the “better ear” effect). 

Thus far, only one study has investigated a similar hypothesis in bimodal patients with hearing preservation (users of electric–acoustic stimulation (EAS)) [15]; however, the study failed to confirm the idea using conventional tests for squelch and redundancy. The recent availability of a number of cochlear implantees having a normal hearing in the non-implanted ear and LF hearing in the implanted ear has allowed us to test this hypothesis more strongly. These patients usually meet the generally acceptable audiometric criteria for SSD cochlear implantation because their pure-tone average (PTA) air-conduction thresholds (for 0.5, 1, 2, and 4 kHz) in the implanted ear is 90 dB or greater, and the PTA in the non-implanted ear is usually no worse than 30 dB.

Due to the rarity of the population of CI users with SSD and preserved residual hearing in the implanted ear, the sample size in the current study is small. A within-subjects, repeated-measures design was used to compare the performance of the patients when the LF hearing in their implanted ear was unmasked against the performance when it was masked. In testing the hypothesis as to whether hearing preservation in the implanted ear provides extra hearing benefits (via squelch and redundancy, over and above the “better ear” effect), we investigated all three binaural effects. 

The difference in performance between masked and unmasked conditions will indicate the level to which preserved LF hearing facilitates access to bilateral cues, mainly ITD_fine_. It has been shown that some EAS users (bimodal patients with hearing preservation) exhibit sensitivity to ITD_fine_ for low-frequency stimuli [9,16]. In this population, the extent of sensitivity to ITD_fine_ can be affected by two factors—one related to hearing preservation and the other to the nature of the sensorineural hearing loss. In the implanted ear, electrode insertion trauma and the presence of the electrode in the cochlea can reduce fine time sensitivity. In the contralateral ear, HL alone can also compromise the extraction of fine structure information. 

Unlike the EAS population studied by Gifford et al. [9,16], the sensitivity of our SSD CI patients to interaural time differences (ITD_fine_) depended only on preservation of the fine time structure mechanism in the implanted ear (since they had full access to fine structure cues through NH in the non-implanted ear).

We also aimed to measure the relationship between the benefit gained from the squelch effect and the patient’s unaided localization ability (in which patients use preserved low-frequency hearing on the CI side, with CI switched off, and NH from the contralateral side). Our rationale was that when a patient listens via preserved LF hearing in one ear and NH in the other, localization will be based mainly on ITD_fine_ and minimally on the interaural level difference (ILD), as indicated by previous studies [15,17,18]. This rationale is in line with the finding that high-frequency hearing loss may prevent hearing-impaired listeners from using high-frequency-dominated ILDs and spectral cues [19,20,21]. Our study population relied on ITD_fine_ since both unaided localization ability and the mechanism underlying the benefit from the binaural integration effects of squelch depend on this factor. Any inability to effectively use ITD_fine_ cues (possibly due to a lack of fine structure mechanism in the LF hearing of the implanted ear) will have a negative impact on both localization ability and squelch. Therefore, we conclude that measured unaided localization ability should serve as a reliable indicator of the ITD_fine_ available to these patients through the preserved LF hearing in the implanted ear. Finally, we decided to test if there was a relationship between thresholds of preserved LF hearing, the level of benefit from binaural integration effects, and localization ability. To our knowledge, no previous studies have investigated binaural effects in SSD CI patients with preserved residual hearing in the implanted ear.

## 2. Materials and Methods

A group of 11 adult CI users with preserved low-frequency hearing in the implanted ear, and with normal hearing or mild hearing loss in the contralateral ear, were included in the study. All patients fulfilled the criteria of single-sided deafness (SSD) [6].

**Figure 1:**
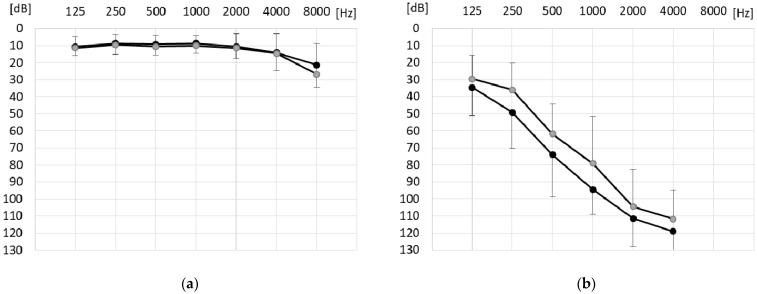
Mean preoperative (grey circles) and postoperative (black circles) audiometric thresholds (*n* = 11) for (**a**) non-implanted ears and (**b**) implanted ears. Whiskers show standard deviations.

Patients were implanted with MED-EL devices at the Institute of Physiology and Pathology of Hearing, Poland, in their poorer-hearing ear using the six-step round-window approach surgery developed by Skarzynski et al. [22]. The round window approach minimizes trauma to the delicate cochlear structures because it does not require drilling of the cochlea’s bony capsule and thus avoids the risk of acoustic trauma and incorporation of bone dust. If needed, an anterior tympanotomy is performed for better visualization of the round window membrane. The anterior tympanotomy is inherent to the endomeatal technique, which can be considered as an alternative approach in some anatomically challenging cases [23] In all patients, except one, short electrodes were used (details of types of electrodes are presented in Table 1). Mean audiometric preoperative and postoperative thresholds are given in Figure 1. Pure-tone average (PTA) air-conduction thresholds (for 0.5, 1, 2, and 4 kHz) for the implanted ear, and the ear contralateral to the CI are also presented in Table 1 for each patient. For the implanted ear, PTA thresholds were calculated on the basis of at least two measurable thresholds out of four required, and if there were missing values, the maximum output of the audiometer (130 dB HL) was used for the calculation.

All patients fulfilled 14 months of CI follow-up. The age at CI ranged from 18 to 61 years old (mean 42; SD 13.9). Patient demographic data are presented in Table 1. Following surgery, the speech processor was fitted for each subject according to the CI manufacturer’s guidelines. An FS4 speech coding strategy was used for all patients. 

Binaural effects in the study group were evaluated with the Polish monosyllabic word test. The test was performed in an anechoic chamber for three different spatial locations of speech and noise to evaluate possible binaural effects. Binaural redundancy was evaluated by presenting words alone or words plus noise from a loudspeaker in front of the subject. The squelch effect was evaluated by presenting words from a loudspeaker in front of the subject and noise at 90 degrees from the midline to the implanted ear. The “better ear” effect was evaluated by presenting words from a loudspeaker on the subject’s implanted side and noise from a loudspeaker on the subject’s non-implanted side; the loudspeakers were set at 90 degrees from the midline. The noise was speech-spectrum noise at 60 dB SPL and was played continuously. Each subject was first tested in each listening setup with their CI deactivated. To maximize the sensitivity of subsequent measures and avoid floor or ceiling effects, the presentation level of the words was adjusted until a score of around 50% correct was achieved. Then, each subject was tested with their CI using the words at an adjusted level. The procedure is described in detail in Lorens et al. [1].

The sound localization test was performed in an anechoic chamber using a custom-made system of 11 loudspeakers (Indiana Line Nano 2) arranged in a semicircle 2 m in diameter in the frontal horizontal plane; the loudspeakers, hidden behind a curtain, were separated by 10° and ranged from −50° (left) to 50° (right). Subjects were seated at the center of the semicircle. With the use of a control pad, the listener lit up one of 141 LEDs (separation 1°) located underneath the loudspeakers from −70° (left) to 70° (right) corresponding to the direction that they perceived the sound to come from. For the localization test, 11 different environmental sounds were used. The sounds were presented randomly twice per loudspeaker. The localization test procedure is described in detail in Skarzynski et al. [3]. The patients’ sound localization ability was tested in the unaided condition, that is, via preserved hearing in the implanted ear and normal hearing in the other.

To evaluate the contribution of preserved low-frequency hearing in the implanted ear to binaural integration, patients were tested in two listening configurations—unmasked and masked. The unmasked configuration allows the patient to access the temporal fine structure of the signal via preserved hearing in the implanted ear and via normal hearing in the other (which we call the low frequency/normal hearing (LF–NH) configuration). A second listening setup was configured to mask the temporal fine structure in the implanted ear but not in the ear with normal hearing, which is designated the low frequency masked/normal hearing (LF_masked_–NH) configuration. For this purpose, the implanted ear was plugged, and the earphone transmitted white noise. Both listening configurations were tested twice—once with the CI switched on and again with it off. The contribution of preserved residual hearing to binaural effects was evaluated by comparing the benefits from both listening configurations (LF–NH and LF_masked_–NH). The binaural benefit of the CI was calculated twice (for masked and unmasked listening configuration) as the difference between the “CI off” and “CI on” conditions for each listening setup (redundancy, squelch, and “better ear” effect).

To answer the research question of whether unaided sound localization ability is an indicator of the availability of ITDfine, the relationship between binaural benefits and unaided sound localization ability was investigated. In addition, the relationship between binaural benefits and the range of preserved hearing in the implanted ear (PTA_[125–500]_ calculated for 125, 250, and 500 Hz), and between unaided sound localization ability and PTA_[125–500]_, were examined.

For both conditions (LF–NH and LFmasked–NH), a Student’s t-test was used to make pair-wise comparisons of speech outcomes in “CI off” and “CI on” conditions for each test setup (redundancy, squelch, and “better ear” effect). The hypothesis of a normal distribution of the data was evaluated using a Shapiro–Wilk test. The relationships between binaural benefits and unaided sound localization error, and between those and PTA_[125–500]_ in the implanted ear, were analyzed by means of Pearson correlations. Pearson correlations were also used to investigate relationships between unaided sound localization error and PTA_[125–500]_ in the CI ear. Values of *p* < 0.05 were considered statistically significant.

## 3. Results

**Figure 2:**
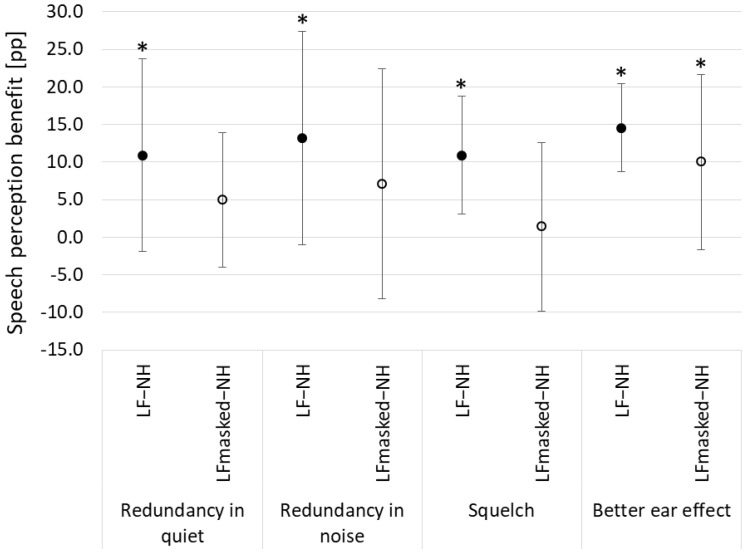
Speech perception benefit for different test setups (redundancy in quiet; redundancy in noise; squelch, and “better ear” effect) and two listening configurations (low-frequency hearing in implanted ear masked, (LF_masked_–NH); low-frequency hearing in implanted ear unmasked (LF–NH)). Symbols mark mean benefit; whiskers show standard deviations; asterisks indicate significant differences.

For the LF–NH configuration, pair-wise comparisons of the “CI off” and “CI on” conditions showed significant differences for redundancy in quiet (*t*(10) = 2.8; *p* = 0.02), redundancy in noise (*t*(10) = 3.1; *p* = 0.01), squelch (*t*(10) = 6.2; *p* = 0.0001), and “better ear” effect (*t*(10) = 6.1; *p* = 0.0001). For the LF_masked_–NH configuration, pair-wise comparisons of the “CI off” and “CI on” conditions showed significant difference only for the “better ear” effect (*t*(10) = 3.0; *p* = 0.01). Figure 2 shows the differences (for masked and unmasked listening configuration) between “CI off” and “CI on” conditions for each listening setup separately.

**Figure 3:**
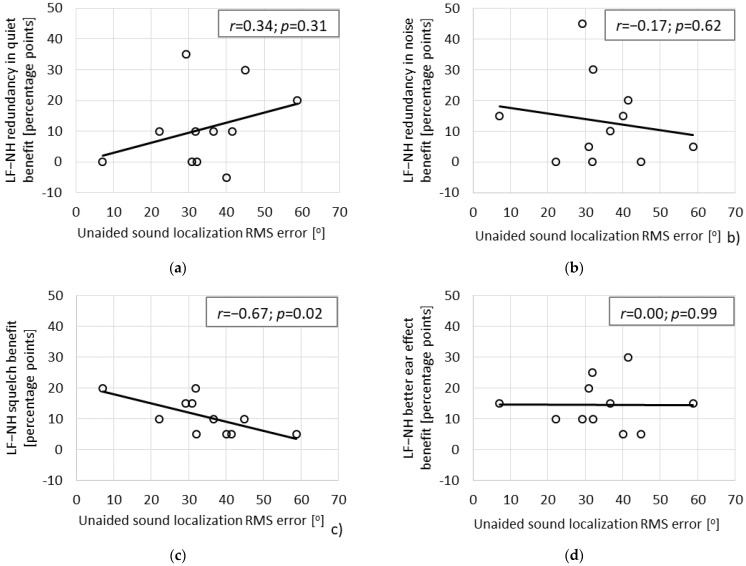
Pearson correlation between various forms of binaural benefit (*y*-axis) (**a**) redundancy in quiet, (**b**) redundancy in noise, (**c**) squelch, and (**d**) better ear effect and unaided sound localization error (*x*-axis) (*r* = correlation coefficient; *p* = significance level).

Figure 3 shows the Pearson correlations between binaural benefits in the LF–NH listening configuration and unaided sound localization error. A significant negative correlation (*r* = −0.67; *p* = 0.02) was found only for squelch (Figure 3c). There was no correlation between binaural benefits in the LF–NH listening configuration and PTA_[125–500]_ in the CI ear. The Pearson correlations and significance levels were: *r* = 0.26, *p* = 0.45 for redundancy in quiet; *r* = 0.03, *p* = 0.93 for redundancy in noise; *r* = 0.42, *p* = 0.20 for “better ear” effect; and *r* = −0.28, *p* = 0.41 for squelch. There was also no correlation between unaided sound localization error and PTA_[125–500]_ in the CI ear (*r* = 0.48, *p* = 0.14).

## 4. Discussion

### 4.1. Contribution of Preserved LF Hearing to Integration Benefit of Redundancy and Squelch

An interesting aspect of our study is that the patients differed significantly from those tested in previous studies. Our patients were unique in the way they had successful hearing preservation surgery in one ear and normal or close to normal hearing (NH) in the other ear. Thus, we were able to explore the value of preserved LF hearing in the implanted ear for binaural integration effects of redundancy and squelch, assuming that all binaural cues are available from the NH ear. Generally, when the “CI on” condition was compared to the “CI off” condition, the average level of speech perception improved for redundancy in quiet, redundancy in noise, “better ear” effect, and squelch in the unmasked condition (although not in the masked condition). When preserved LF hearing in the implanted ear was masked, the only significant improvement was for the “better ear” effect. Hence, we have confirmed our hypothesis that bilateral acoustic LF hearing, which is available thanks to hearing preservation in the implanted ear, provides extra benefits via squelch and redundancy effects (over and above the “better ear” effect).

The finding that in SSD CI patients the benefit provided by a CI, without the contribution of LF hearing in the implanted ear, reflects a “better ear” advantage effect is in line with most previous studies [4,24,25,26,27,28]. There are at least two possible reasons why conventional SSD CI patients derive very little benefit from the binaural integration effects of redundancy and squelch. Firstly, the signal processing performed by most commercially available CIs removes most of the fine timing information present in a sound. These CIs convey temporal information only in the modulated envelope of sounds (ITD_env_) since stimulating electrical pulses are presented at a fixed, high rate completely unrelated to the sound’s temporal fine structure. Uniquely, however, to enhance temporal information within the electrical stimulation signal, CIs manufactured by the MED-EL company lower the stimulation rate of the low-frequency channels and alter the timing of the pulses to fire at times of zero-crossing of the corresponding band’s signal. Secondly, there is commonly a mismatch between the place of excitation in one ear and in the other. The electrode arrays produced by most CI companies are not fully inserted into the cochlea (an exception being those manufactured by MED-EL). For a low frequency narrowband acoustic input signal, in particular, the CI will stimulate a certain electrode in the cochlea according to its frequency-to-electrode location, which for shallower insertion will not correspond to the cochlear frequency-to-place map of normal hearing. Thus, for SSD CI patients, different places in the two cochleas will usually be stimulated, which will degrade ITD_fine_ cues [29,30].

However, our observation that in the masked condition, there were no redundancy and squelch effects should be interpreted with caution. Firstly, the LF acoustic masker could, theoretically, also mask the fine time structure of the signal included in the electrical stimulation. Secondly, in the majority of our patients, 24-mm electrodes were used, which does not give deep insertion. Thirdly, the low cut-off frequency was programmed according to the EAS fitting rules, a frequency higher than in regular patients, and hence, the coding strategy that could have provided access to the temporal fine structure of the signal at LF was not fully available. In summary, our patients were at a disadvantage for accessing temporal fine structure through their CI. On the other hand, it needs to be mentioned here that many regular patients, through similar reasons, are at the same disadvantage as far as temporal fine structure is concerned.

Our data show that SSD patients with hearing preservation benefit from the binaural integration effects of redundancy and squelch, in addition to the “better ear” effect. However, when preserved LF hearing in the implanted ear was masked, the binaural benefit was reduced to the “better ear” effect. As binaural effects in these patients have not been previously investigated, our results may be somewhat different from those on the contribution of preserved LF hearing conducted in different populations.

Contrary to our findings, Gifford et al. [15] have demonstrated that bimodal listeners with hearing preservation (EAS users) failed to exhibit binaural integration benefits, as revealed by redundancy and squelch effects. According to the authors, a possible reason for the lack of binaural effects in the hearing preservation patients was the heterogeneity of the electrode arrays. Some patients in their study were implanted with either Nucleus perimodiolar arrays or Sonata standard electrodes, both of which are not designed with hearing preservation in mind. In the current study, purportedly atraumatic electrodes were used, which may facilitate hearing preservation in terms of thresholds and in terms of preserving structures responsible for fine time coding. Our study group apparently had access to the fine structure of the signal; the preserved LF hearing facilitated the availability of ITD_fine_ and gave binaural integration benefits. Another possible reason for the difference in outcomes between the current study and that by Gifford et al. [15] could be in the populations themselves. In our study, the SSD CI patients had full access to the fine structure of the signal in the ear contralateral to the implant due to NH in that ear. In the study by Gifford et al. [15], however, the bimodal patients with hearing preservation had considerable hearing loss (HL) in the non-implanted ear—they had a wide range of LF thresholds varying from 10 dB to 80 dB and had high-frequency hearing thresholds worse than 80 dB. Thus, contrary to our study population, the access of the EAS patients to fine structure might also have been compromised by HL in the non-implanted ear. There is ample evidence that hearing impairment adversely affects the ability to use temporal fine structure cues [31,32,33,34]. Moreover, Lorenzi et al. [35] have demonstrated that for people with hearing loss at medium to high frequencies, processing of temporal fine structure in speech can be degraded at LF, even when absolute thresholds at those frequencies are within the normal range.

In addition, there is also a study showing that preserved LF hearing does not appear to contribute to the redundancy effect, a finding that is again not in line with our study. Sheffield et al. [36] tested bimodal patients with hearing preservation for monosyllabic word discrimination in multi-talker babble noise, in which speech and noise were both presented from the front. There was no difference in speech discrimination between the two listening conditions—CI + non-implanted acoustic hearing (bimodal) and CI + bilateral acoustic hearing (bimodal with hearing preservation)—indicating there was no additional speech benefit from preserved LF hearing in the implanted ear. In the current study, we observed a significant redundancy effect in the unmasked condition (which is related to a configuration of bimodal hearing with hearing preservation), but we did not see it in the masked condition (which relates to the bimodal configuration). This finding suggests that there is an additional speech benefit from having preserved LF hearing in the implanted ear (as measured in the redundancy test).

The results of the current study can also be compared to findings from the simulation study of Williges et al. [13]. In this study, configurations of (1) unilateral and bilateral CIs, (2) unilateral and bilateral CIs with hearing preservation in the implanted ear, and (3) bimodal listening were simulated by presenting NH listeners with different combinations of vocoded and low-frequency narrow-band speech. A squelch benefit was found when bilateral CIs with LF hearing preservation was simulated, and also when a unilateral CI with LF hearing preservation was combined with LF hearing in the contralateral ear. When unilateral and bilateral CIs without LF hearing preservation were simulated, the results showed bilateral squelch close to zero. The simulations of Williges et al. [13] indicate that CI users with additional low-frequency bilateral acoustic hearing show a binaural benefit from squelch, in contrast to CI users with no acoustic hearing. The authors conclude that bilateral CI users with LF hearing preservation, and unilateral CI users with hearing preservation and LF hearing in the contralateral ear, do appear to have ITD_fine_ cues available by virtue of LF acoustic hearing. When speech and noise are spatially separate, these ITD_fine_ cues benefit speech understanding.

Sensitivity to low-frequency ITDs in bimodal patients with hearing preservation was demonstrated by Gifford et al. [9,16]. Results revealed that, for most bimodal listeners with hearing preservation, thresholds of interaural time differences (ITD) carried by a low-frequency, bandpass noise (100–800 Hz) were within the physiologically relevant ITD range. At the group level, no significant effects of unilateral electrical stimulation on the resultant ITD thresholds were observed [16].

### 4.2. Relation between Binaural Effects and Localization Ability

Having in mind that IDT sensitivity is possible in CI patients with hearing preservation, we decided to look at how the binaural benefits of redundancy, squelch, and “better ear” effect depended on unaided localization ability. We assumed that if localization ability and the mechanism underlying the benefit from squelch effect both rely on the same ITD_fine_ cues, then those SSD patients with bilateral LF acoustic hearing who have good localization ability were also more likely to benefit from the squelch effect, but not from redundancy or “better ear” effects since these other effects do not depend so much on ITD cues. Our results confirm this assumption.

Figure 3 summarizes the results of correlation analyses for binaural effects and unaided localization ability. It shows that there is a significant negative correlation between CI benefit via a squelch and unaided localization error. However, there is no correlation between redundancy and localization error or between the “better ear” effect and localization error due to the fact that, as already mentioned, a redundancy effect and a “better ear” effect do not rely on ITD_fine_ cues. Ching et al. [37] have demonstrated a redundancy effect in adult bimodal patients, although these patients were unable to use ITD cues. Thus far, as far as the “better ear” effect is concerned, it requires a spatial arrangement of speech and noise so that the CI ear receives a more favorable SNR. In masked and unmasked conditions, our SSD CI patients could take advantage of this SNR difference by selectively attending to the CI ear.

Another interesting observation in the current study was that the binaural integration effect of squelch depends on factors other than audibility. That is to say, squelch and its binaural integration effect is negatively correlated with localization error, although not with the hearing threshold of preserved LF hearing (PTA_[125–500]_). This finding provides additional evidence for the idea that implant recipients with preserved acoustic hearing have continued access to fine structure cues, which should provide greater binaural integration benefits. Additionally, we found there was no correlation between localization ability in the unaided condition and the level of LF hearing (PTA_[125–500]_). Since both the binaural integration effects of both squelch and localization rely on sensitivity to ITDfine cues, the lack of a significant correlation between squelch and PTA_[125–500]_, and between localization ability and PTA_[125–500]_, suggests there is no relationship between access to ITD_fine_ and the level of LF hearing. This agrees with Strelcyk and Dau [38], who, for a test frequency of 750 Hz, found no correlation between ITD sensitivity and hearing threshold. It also supports the findings of Hopkins and Moore [39], who found only a weak correlation at 750 Hz and no correlations at 500 and 250 Hz.

Since SSD CI patients have NH in the non-implanted ear, their better access to fine structure in the implanted ear should translate into greater access to ITD_fine_ cues. Therefore, as demonstrated by our study, those patients who show improved localization abilities when listening via LF hearing in one ear and NH in the other (a task based on ITD_fine_), are also more likely to receive benefits from binaural squelch. According to our testing paradigm, the unaided localization test score in those patients who have CI surgery with hearing preservation can be seen as a marker of LF fine time structure coding. Nevertheless, the level of fine structure accessible to a patient cannot be simply deduced from the hearing thresholds measured postoperatively, which is a conclusion supported by (1) the lack of correlation between CI benefits via a squelch effect in the LF–NH listening configuration and the level of PTA in the implanted ear and (2) the lack of correlation between unaided sound localization error and the level of PTA in the implanted ear.

### 4.3. Clinical Relevance

When discussing clinical relevance, there are two aspects to consider. Firstly, because the indication criteria for cochlear implants have progressively been extended, the population of CI candidates has increased in range and type of hearing loss [40]. Patients with residual LF hearing in the ear to be implanted and SSD patients are two patient groups that have recently been identified as warranting attention in any discussion of CI candidacy [41]. Our study has investigated a third group of CI candidates, i.e., individuals who, according to audiometric stratification of types of hearing loss, have SSD although at the same time possess an LF residual hearing in the ear opposite to the NH ear. These patients have thus far not been discussed as CI candidates. However, the results of the current study indicate that these patients can benefit from a CI through binaural integration effects and via a “better ear” effect. Residual LF hearing in the ear to be implanted should not be considered as a contraindication for a CI; instead, if the LH hearing is preserved, this makes the candidate open to additional hearing benefits via the squelch and redundancy effects (over and above the “better ear” effect typically observed in an SSD patient without LF hearing in the implanted ear).

Secondly, due to the large variance in the binaural benefits of SSD CI patients, there is a need for appropriate clinical tests that can be used shortly after implantation to predict possible CI-related benefits that might be achievable in the course of auditory rehabilitation. Results of the current study suggest that in SSD patients with LF hearing preservation, the localization error obtained in the unaided condition can serve as a predictor of the binaural integration effect of squelch and, more generally, of the possible hearing benefits, which stem from accessibility to fine time structure from LF hearing in the implanted ear. The open question is whether this finding can be generalized to other populations of patients with hearing preservation who are EAS users (that is, bimodal listeners with hearing preservation). Future investigation is needed to verify that acoustically based localization ability in EAS users can be used as a marker of sensitivity to fine time structure in the implanted ear and therefore indicates the potential for increased benefits from the integration effects of redundancy and squelch.

## 5. Conclusions

The findings of the current study are potentially good news for SSD patients who, after fulfilling the criteria for a CI, end up with preserved LF hearing in the operated ear and NH in the contralateral ear. Our results indicate that it is possible for them to have access to the binaural integration effects of redundancy and squelch and receive additional speech understanding in noise. To our knowledge, the results here provide the first clinical evidence that preserved low-frequency hearing contributes to binaural integration effects of redundancy and squelch, presumably by allowing access to ITD_fine_ cues. In any particular case, the extent of the improvement does not depend greatly on the threshold of the preserved LF hearing. It seems that in these CI patients, the benefit obtained from squelch is related more to localization error, as measured in the unaided condition. We, therefore, conclude that localization error might serve as a good predictor of the temporal fine structure cues that preserved hearing can provide.

In brief, hearing preservation after cochlear implantation is beneficial since it preserves low-frequency hearing in the implanted ear, and this can contribute to binaural integration.

## Figures and Tables

**Table 1:** Subject data (f = female; m = male; SIHL = sensorineural idiopathic hearing loss; CI = cochlear implant; SSD = single-sided deafness).

No.	Gender	Etiology	Hearing Loss Type	CI Type	Electrode Type	Processor Type	EAS System	CI Ear	Type	Post-Op PTA Non-CI Ear [dB]	Pre-Op PTA CI Ear [dB]	Post-Op PTA CI Ear [dB]	Tinnitus before CI?	Duration of Deafness [years]	Age at CI [years]	Extent of CI Use [hours/day]
1	m	head trauma	sudden	Sonata	Flex 24	Sonnet	yes	right	ssd	9	80	111	yes	2	33	10
2	m	SIHL	sudden	Sonata	Flex 20	Sonnet	yes	left	ssd	9	63	74	yes	12	61	14
3	f	otosclerosis	progressive	Synchrony	Flex 20	Sonnet	no	left	ssd	20	80	103	yes	2	50	16
4	m	head trauma	sudden	Sonata	Flex 24	Sonnet	yes	right	ssd	5	108	105	yes	3	23	12
5	m	unknown	sudden	Concerto	Flex 20	Sonnet	yes	right	ssd	5	64	68	yes	13	35	16
6	f	unknown	sudden	Sonata	Medium	Opus2	no	left	ssd	13	115	115	yes	31	53	10
7	f	SIHL	sudden	Concerto	Flex 24	Rondo	no	left	ssd	11	96	116	yes	6	56	16
8	m	after virus infection	sudden	Concerto	Flex 24	Rondo	no	left	ssd	5	103	114	no	7	18	12
9	m	unknown	sudden	Sonata	Flex 24	Sonnet	yes	right	ssd	4	94	104	yes	4	40	12
10	f	unknown	sudden	Concerto	Flex 24	Sonnet	yes	right	ssd	25	76	104	yes	3	52	16
11	f	unknown	sudden	Sonata	Flex 28	Sonnet	no	left	ssd	11	105	106	yes	11	46	10

## Data Availability

The data that support the findings of this study are available from the corresponding author, upon reasonable request. The data are not publicly available due to legal restrictions (they contain information that could compromise the privacy of research participants).
